# Free energy of collagen-mimetic peptide dimerization and implications for fibrillization

**DOI:** 10.1016/j.bpj.2026.01.010

**Published:** 2026-01-13

**Authors:** George A. Pantelopulos, Ayan Majumder, John E. Straub, Robert B. Best

**Affiliations:** 1 Laboratory of Chemical Physics, National Institute of Diabetes and Digestive and Kidney Diseases, National Institutes of Health, Bethesda, Maryland; 2 Department of Chemistry, Boston University, Boston, Massachusetts

## Abstract

Proper assembly of collagen fibrils is essential, as they constitute a plurality of protein mass and structure in extracellular matrices. However, the molecular determinants of the collagen fibrillization mechanism are difficult to characterize, in part due to the size and heterogeneity of the collagen triple helix. We have used MD simulations to characterize the dimerization free energy landscape of model collagen-mimetic peptide triple helices. Under in vivo buffer conditions, we find that domains consisting purely of proline-hydroxyproline-glycine (POG) repeats readily dimerize via a tight hydrophobic association stabilized via additional hydrogen bonds involving hydroxyprolines (Hyp). For a model heterotrimeric triple helix optimized for stability using salt bridges, we find a much weaker association free energy minimum between triple helices. Notably, interstrand salt bridges within each triple helix do not readily break upon the encounter of two triple helices, and these longer side chains also block hydrophobic and Hyp-Hyp hydrogen-bonded interactions. In contrast, we find that a “charge zipper” sequence, designed to avoid intrahelix and promote interhelix salt bridges, forms dimers that are more than twice as stable as associations of POG-repeat triple helices. These results reveal that there are multiple modes of association of collagen triple helices that appear, to a large extent, orthogonal. Analysis of fibrillar collagens shows that, whereas charged residues are typically expected to drive fibrillization, approximately one-fourth of charged residues are involved in salt bridges within triple helices and may effectively be unavailable for participation in helix-helix interactions and interactions with other proteins in extracellular matrices.

## Significance

Collagen is the single most abundant type of protein in mammals and a major component of the extracellular matrix. Because of its important physiological role and the involvement of collagen mutations in many diseases, it is important to understand the mechanism by which collagen triple helices self-assemble into fibrils. We have studied three small model systems that shed light on the molecular interactions that can stabilize collagen dimers. Our work shows multiple possible modes of interaction, involving either shorter-range hydrophobic interactions or longer-range salt-bridge interactions. We also show that there is a frustration between intra- and intermolecular salt bridges that needs to be considered when modeling charge interactions between helices.

## Introduction

**Figure 1:**
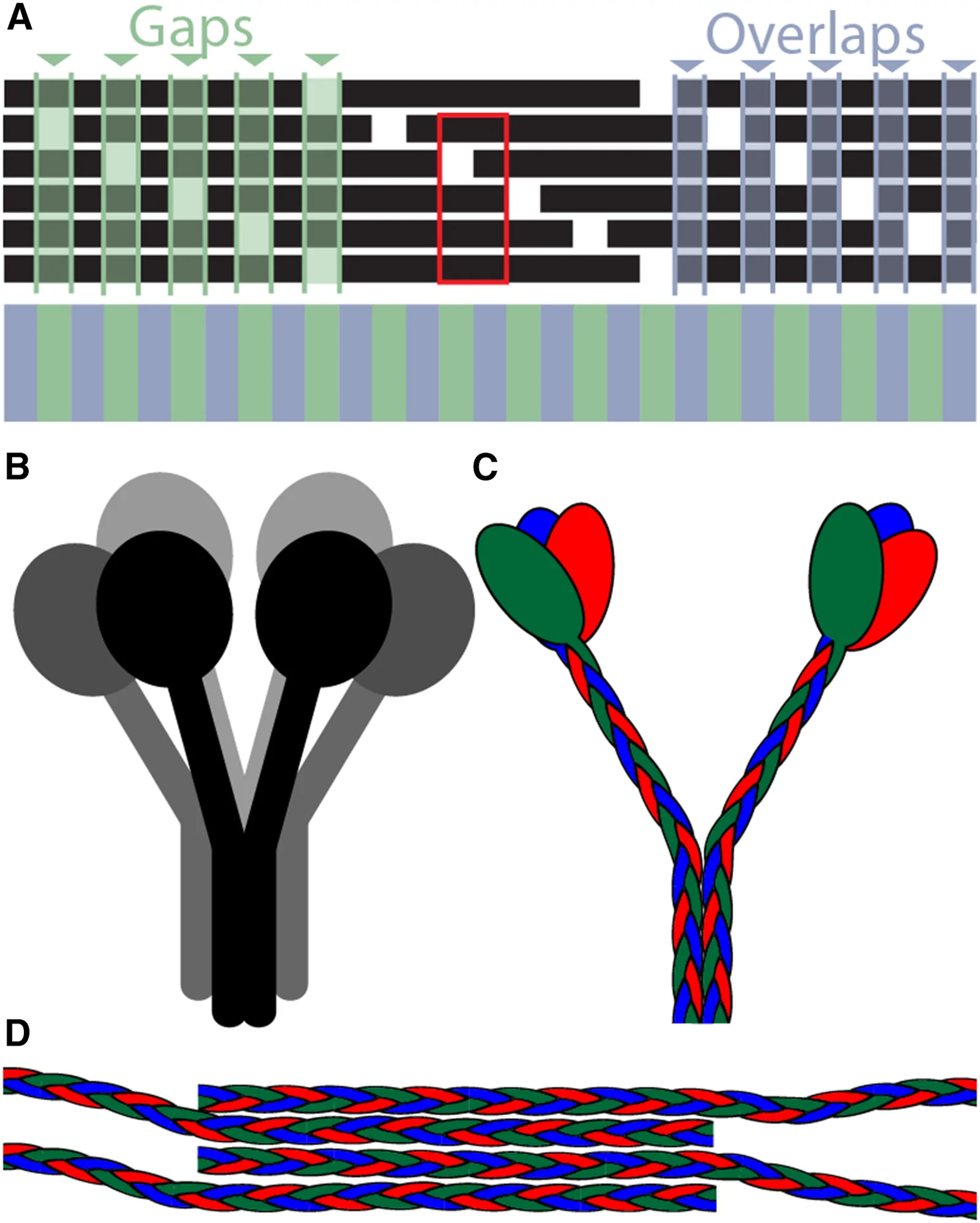
Interactions between collagen triple helices exist in several contexts. (*A*) Petruska and Hodge-like 2D model of the arrangement of collagen XYG-repeat domain triple helices (*black bars*) in the collagen fibril (9). Overlaps (*blue*) are higher-density space featuring flush helix-helix interactions. Gaps (*green*) are lower-density space partially occupied by the less ordered non-XYG-repeat telopeptides at N- and C-terminal domains. The red rectangle represents the periodic unit of the fibril. The resultant alternating *D*-periodic barcode-like pattern of higher and lower electron density observed in electron micrographs is illustrated. (*B*) Cartoon of hexameric oligomer of collagen triple helices of defense collagens, showing globular domains as ellipses at the C-termini. (*C*) Cartoon depicting the triple-helical domains of the metastable defense collagen adiponectin dimer with individual protein strands in blue, green, and red. (*D*) Cartoon depicting the collagen IV N-terminal 7S domain tetramer with individual protein strands in blue, green, and red.

Collagen fibers are ubiquitous in mammalian extracellular matrices (1,2,3,4). The structure and mechanics of collagen fibrils have been investigated for decades, leading to some universal conclusions about collagen fibril structures and sequences. Among these are the observation that functional collagen fibrils manifest a characteristic ∼67-nm-long *D*-banded structure, featuring periodic bands of ∼0.4*D*-long “gap” regions of lower electron density and ∼0.6*D*-long “overlap” regions of higher electron density (5,6), with variations of a few nanometers possible depending on collagen type and solution condition (7). These gap regions correspond to positions along the principal axis of the fibril, at which there are approximately 4/5 atoms from triple helices present, compared to overlap regions. This observation led to the development of theoretical models of collagen fibrils built upon the idea that the periodic unit of the fibril must consist of five parallel collagen triple helices, within which 1/5 of each collagen triple helix is present, ultimately containing subdomains of one full collagen triple helix (8) (Fig. 1 *A*).

The current atomistic model of collagen fibril was determined via X-ray diffraction by Orgel et al. (10) at a low, ∼1-nm resolution. This model has served as the basis for physics-based modeling of a collagen fibril structure with fully explicit solvent molecular dynamics (MD) simulations by Obarska-Kosinska et al. and Monego et al. (11,12). There are also examples of other, lower-order protein assemblies featuring the characteristic Xaa-Yaa-Gly repeat triple helices, which define collagenous domains in biology. For example, the defense collagens, such as adiponectin and C1q (Fig. 1, *B* and *C*), have been observed to form oligomers (13), such as between the “stem” domains of C1q, which form a hexamer (14) whose structure was recently solved by cryoelectron microscopy (15), or in assemblies as small as dimers stabilized by disulfide bonds, as has been observed for adiponectin (16). The collagen VI microfibril features specific interactions between pairs of triple helices in parallel and antiparallel alignments (17,18). Collagen IV features the lengthy 7S domain, which forms a tetramer of parallel and antiparallel triple helices (19,20) stabilized via disulfide bonds (Fig. 1 *D*), which initially assembles via noncovalent helix-helix interactions and may involve a metastable dimeric state (21). There remains much to be done to elucidate the details of residue-residue interactions necessary for nucleating fibril formation and stabilizing the fibril in its characteristic *D*-periodic structure, in addition to other collagen-collagen interactions featuring the triple helix.

Collagen mimetic peptides (CMPs), which feature the Xaa-Yaa-Gly repeat motif, have been widely used to investigate variations in structure, stability, and mechanics across various sequences. CMP investigations have typically employed a “host-guest” approach, using the proline-4(R)-hydroxyproline-glycine (Pro-Hyp-Gly [POG]) repeat motif as a “host,” as this repeat appears frequently in fibrillar collagens and maximizes the thermal stability of the triple helix due to its preference for configuring into the polyproline II secondary structure, which each strand occupies in the triple helix (22,23). CMP “guest” substitutions in the POG repeat are typically made using solid-phase synthesis to lengths of 24–42 residues, maintaining (POG)_3_ or (POG)_4_ caps at the N- and C-terminal ends of the peptides to stabilize the triple helix. Crystal structures of CMPs have confirmed that the collagen triple helix is stabilized by one amide and two aliphatic hydrogen bonds involving glycines appearing at every third residue (24,25), such that the substitution of Gly with any other amino acid dramatically hampers folding of the triple helix and appears in many single-point mutations implicated in tissue diseases (26). It has also been discovered that the triple helix can be stabilized through the formation of salt bridges and cation-*π* interactions between side chains of strands in the triple helix (27,28,29). This has led to the engineering of CMP designs that can be highly enriched in intrahelix salt bridges, which can manifest a triple-helix thermal stability approaching that of POG-repeat triple helices (30,31), of which the sequences designed using the SCEPPTr scoring function are perhaps best known (30). Interhelix salt bridges have also been employed to drive the assembly of CMP fibrils with a short *D*-band (32,33,34). A dimerization constant has yet to be determined for any pair of CMPs experimentally due to the propensity for most CMPs to readily self-assemble into fibril-like structures (35) or oligomers (15). There has yet to be evidence for a CMP that forms a dimeric state that can be experimentally characterized using a conventional method. However, a kinetic model of oligomerization and fibrillization would necessarily rely upon a dimerization free energy term and is thus a central quantity of interest for understanding both collagen oligomerization and fibrillogenesis.

**Figure 2:**
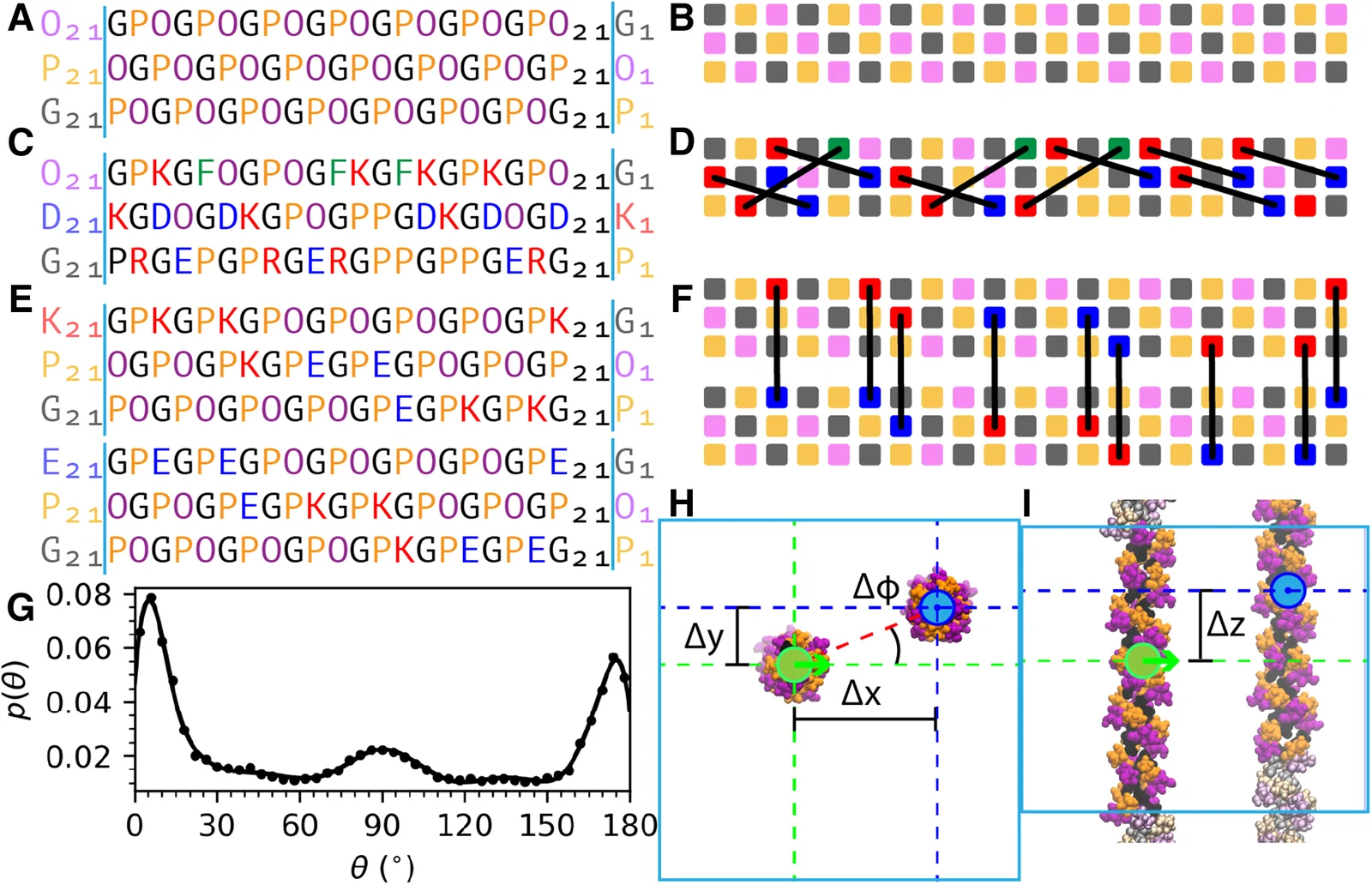
Triple-helix pairs considered in this work. (*A* and *B*) POG-repeat and (*C* and *D*) SCEPPTr-designed triple helices made periodic through the periodic boundary condition (*light blue*), of which parallel and antiparallel configurations are tested. Pro, Hyp, and Gly are in orange, purple, and black, respectively. Positively charged residues (Lys and Arg) are in blue. Negatively charged residues (Asp and Glu) are in red. Aromatic residues (Phe) are in green. (*E* and *F*) Sequences of the two different triple helices used to form the charge zipper triple-helix heterodimer, simplified to the color scheme in (*A*) and (*B*). (*B*), (*D*), and (*F*) are simplified representations of the four periodic triple helices for visualizing side chain-side chain interactions. (*D*) SCEPPTr-designed sequence, showing intrahelix side-chain interactions used to stabilize the helix in black lines. (*F*) Charge zipper, showing one set of possible charge-charge interactions that could stabilize the dimer of triple helices. (*G*) Likelihood of angle sampled over all frames between the periodic POG repeat and a (POG)_5_ homotrimer in a conventional MD simulation. (*H*) *x*- and *y*-dimension components of the CV used for metadynamics, as well as the azimuthal angular displacement. The green arrow designates the positive *x*-dimension orientation to which the first triple helix is restrained. (*I*) *z*-dimension component of the CV used for metadynamics.

Here, we have used MD simulations to characterize the dimerization of the free energy landscape of POG repeats, a SCEPPTr-designed sequence, and a “charge zipper” model using a metadynamics approach (Fig. 2). We simulated both parallel and antiparallel orientations of POG repeats to investigate differences in the free energy and structure of the dimer, dependent on orientation, and the SCEPPTr-designed sequence to observe if any possible difference in helix-helix interactions might arise, dependent upon parallel or antiparallel orientations. The charge zipper model triple helix featuring long charged side chains intended to form a specific dimer of triple helices was also simulated to investigate the distance dependence of electrostatically driven dimerization at 150 mM salt. By performing long well-tempered metadynamics (WT-metaD) simulations using multiple collective variables (CVs), we were able to characterize the free energy landscape while also resolving multiple metastable states. We have also conducted a bioinformatic analysis of possible salt bridges formed in natural fibrillar collagens using multiple sequence alignments (MSAs) to explore the implications of our characterization of the dimerization of these collagen-mimetic peptides. The results reveal a rich complexity in the binding energy landscapes, with a strong dependence of the binding free energy on peptide sequence and different optimal interaction distances for different types of interactions, which, taken together with our bioinformatic analysis, have novel implications for the role of charged residues in fibrillogenesis.

## Materials and methods

We characterized the free energy landscape of the dimerization of 21-residue periodic collagen triple helices of varying sequences using a WT-metaD approach we previously developed to characterize multiple metastable states present in transmembrane protein dimers (36,37,38) to overcome limitations to determining dimerization free energies sampled in free energy calculations using a single distance-based collective variable (39,40,41), two CVs (42), or other approaches, such as Replica Exchange (7,43,44,45). Similar successful multiple-CV approaches have been employed for accurately determining free energies of dimerization of transmembrane alpha helices using bias-exchange adaptively biased MD (46) and adaptive biasing force (47). It is also possible to use a single CV from an embedding learned from a set of metastable states supplied a priori, though this comes with significant computational cost (37).

### MD

GROMACS 2022.4 (48,49) patched with PLUMED 2.8.2 (50) was used for all MD simulations. The AMBER ff99sbws force field (51,52,53,54), which is used in combination with a TIP4P/2005 (55) water model featuring 1.1×-scale protein-water Lennard-Jones epsilon terms previously developed for accurate simulations of intrinsically disordered proteins, is an excellent force field for the reproduction of the structure and dynamics of POG repeats and performs well in recapitulating the structure of other CMPs (56). We used ff99sbws for all simulations reported here. The 21-residue-long periodic CMP triple helices studied here are constructed from idealized 7/2 triple-helix superhelical parameters, based on the crystal structure of POG repeats, using the same technique employed in our previous work (56). The 7/2 helix periodically repeats exactly every 21 residues, making it possible to perform a simulation of CMPs, provided that we accept that the 51.4° helical twist per every three residues required to form this structure will be maintained by any CMP if we perform an NVT simulation at an appropriate box length for maintaining the corresponding 6-nm helical pitch (Fig. 2) (57). The pdb2gmx program in GROMACS was used to facilitate the proper assignment of bonded terms for the periodic 21-residue CMP strands across periodic boundary conditions (PBCs).

Conventional and WT-metaD simulations were both performed with the same set of MD parameters, generally appropriate for simulations using AMBER force fields in GROMACS. Hydrogen mass repartitioning with a factor of three was applied such that a 4-fs time step could be used (58). All systems were initially minimized using steepest descent and then equilibrated in the NPT ensemble using weak coupling thermo- and barostats (59), with *τ* = 1 and *τ* = 20 ps at 310.15 K and at 1-bar pressure coupling with 4.5 × 10^−5^ bar ^−1^ compressibility, for 2 ns with a 4-fs time step. All bonds were constrained using LINCS (60), and constraints in water were solved using SETTLE (61).

WT-metaD simulations were performed using Intel Xeon E5-2680 v.4 and Intel Xeon E5-2695 v.3 nodes. Each of the 12 replicas run in parallel was assigned two full CPU nodes, performing at 50 ns/day on simulations of approximately 44,000 particles and 7.4 × 7.4 × 6 nm PBC dimensions, varying slightly depending on system and NPT equilibration during preparation for production simulations. A conventional MD simulation of a POG repeat (Fig. 2 *A*) in the presence of a (POG)_5_ homotrimer, totaling 26,636 particles, and cubic PBC was performed, with conventional MD performing at 238 ns/day on a v100× GPU, with nonbonded terms computed on the GPU and bonded terms computed on the CPU, and pair-distance restraints on pairs of C*α* to prevent fraying or unfolding of (POG)_5_.

### WT-metaD

For each of the five systems studied, we ran 12 parallel simulations for which biases are deposited onto a shared biased potential energy surface. We performed WT-metaD simulations of two periodic POG repeats (Fig. 2, *A* and *B*) in parallel and antiparallel configurations, with 12 trajectories each of 4000- and 3500-ns replicas in length. WT-metaD simulations of a periodic triple helix truncated from the Hartgerink group’s first SCEPPTr-designed triple helix featuring K–E salt bridges and R–F cation-*π* triple-helix-stabilizing interactions (Fig. 2, *C* and *D*) in parallel and antiparallel configurations were performed for 3455 and 3500 ns in each of 12 parallel trajectories. We also performed simulations of a pair of periodic triple helices designed to “zip” up via a specific, alternating set of three electrostatic interactions, which we term the charge zipper system (Fig. 2, *E* and *F*), in a parallel configuration for 3947 ns in each of 12 parallel replicas.

To define the collective variables, the displacements in *x*, *y*, and *z* axes are measured between the center of mass of all alpha carbons of each triple helix (Fig. 2, *H* and *I*). To define a reference frame for the configurational space (62), one triple helix is restrained such that it cannot freely rotate around the *z-*axis via the GROMACS PULL code. An angle-axis restraint with a stiff force constant of 2000 kJ/mol/rad^2^ was applied toa reference vector of this tiple helix to orient it along the positive *x*-axis. The reference vector is defined using the Gly10 Cα of the leading chain and the center of mass of the Hyp and Pro Cα of the middle and trailing chains at the same height as the Gly10 C*α.* . The other triple helix is allowed to rotate freely while WT-metaD is performed in the Δ*x*, Δ*y*, and Δ*z* CV space.

We defined Δ*x*, Δ*y*, and Δ*z* in a periodic coordinate system over the PBC. The WT-metaD bias factor is set to 10 at a temperature of 310.15 K. The Gaussian bias height was set to 0.1 kJ/mol, and there was a sigma of 1 Å in Δ*x*, Δ*y*, and Δ*z*. Gaussian biases were deposited every 5 ps. PLUMED was used to compute the final free energy surfaces, both in the set of CVs used to perform the WT-metaD simulations and the free energy surfaces in alternative sets of CVs after a change of bases. This was done to calculate free energy at CVs after a change of basis to new, uniformly spaced coordinates by properly accounting for the Gaussian biases deposited during simulation. The free energy of dimerization as a function of helix-helix distance converged after approximately 2.8 *μ*s of simulation per parallel replica (Fig. S1), and the final free energies in Δ*x* and Δ*y* (integrating over Δ*z*) are shown in Fig. S2.

### Change of basis

The relative free energy of positions on the free energy landscape in the canonical ensemble in terms of CVs ({*s*}), Δ*A*({*s*}), is defined by the underlying probability density of those CVs *ρ*({*s*}), where Δ*A* = −*k*_B_*Tlnρ*({*s*}), where *k*_B_ is the Boltzmann constant and *T* is the temperature. A change of basis in the coordinate system defined by the CVs ({*s*}) to a new coordinate system defined by CVs ({*s*′}) requires that we account for the Jacobian to perform the change of basis, *ρ*({*s*})/*J* = *ρ*({*s*′}), as demonstrated by Boresch and Karplus (63).

Changing the probability density from the Cartesian basis of the CVs used here, Δ*x*, Δ*y*, and Δ*z*, to the cylindrical coordinate basis used for some analyses, Δ*ϕ*, Δ*r*, and Δ*z*, would require us to compute *ρ*(Δ*x*,Δ*y*,Δ*z*)/*J* = $\rho \left(\sqrt{\Delta x^{2}+\Delta y^{2}},\tan^{-1}\left(\Delta y/\Delta x\right),\Delta z\right)$ = *ρ*(Δ*r*,Δ*ϕ*,Δ*z*), where the Jacobian, *J*, is Δ*r*. In practice, we computed the free energy in new CVs using PLUMED to sum the biases deposited over the course of the simulation every 5 ps at a new set of uniformly spaced coordinates in Δ*r* and Δ*ϕ*. It was necessary to account for the Jacobian when computing the probability distribution of the angle of the (POG)_5_ homotrimer principal axis (${\hat{t}}_{POG_{5}}$) with the principal axis of the periodic POG repeat (the positive *z* axis), $\theta =\cos^{-1}\left({\hat{t}}_{POG_{5}}\cdot \left(0,0,1\right)\right)$. The Jacobian of the azimuthal angle is *sin*(*θ*), and thus, we computed *p*(*θ*)/*sin*(*θ*) and renormalized to determine *p*(*θ*).

### MSAs of fibrillar collagens

For the purpose of determining the position of conserved charged residues in the fibrillar collagens I, II, III, V, and XI, we constructed MSAs of the triple-helical domains of each strand: Col1*α*1, Col1*α*2, Col2*α*1, Col3*α*1, Col5*α*1, Col5*α*2, Col5*α*3, Col11*α*1, and Col11*α*2. It can be challenging to produce a good MSA for the triple-helical domain due to the repeating YGX pattern. We were able to make MSAs of hundreds of sequences by first only using nonredundant sequences in which the longest uninterrupted sequence is a ≥900-residue-long YGX … YGX repeat. With these sequences, we performed clustering with Clustal *Ω* in five iterative steps (64), which resulted in a good alignment of most of the triple-helical domain but maintained some gaps that were not resolvable with further iteration. The remaining gaps in the sequence were manually aligned. The final MSAs before and after this final correction are provided in Data S1. Ultimately, the MSAs are 1014–1020 residues in length and 396 to 1047 sequences in depth. The contiguous YGX domains of fibrillar collagens can be slightly longer than this.

To analyze the Lys, Glu, and Asp positions participating in K–E and K–D salt bridges that likely stabilize the triple helix, we assume a triple-helical alignment without overhanging residues for each type of collagen, similar to the approach taken by Malcor et al. (65). With these trimers, we consider the positions where over 80% of the residues are Lys or 80% of the residues are Glu or Asp. Likewise, for analyzing the Lys, Arg, Glu, and Asp positions in the MSA generally available for participating in interprotein interactions and fibrillization, we also consider positions where over 80% of the residues are Lys or Arg or are Glu or Asp and are not in a position to be engaged in axial or lateral salt bridges formed by Lys-Glu or Lys-Asp. We did not consider Arg-Glu or Arg-Asp salt bridges, as these are significantly less stable than the intrahelix salt bridges formed using Lys (66), and Arg is featured in the chaperone HSP47 binding motif necessary for folding the triple helix (67).

## Results and discussion

In this work, we focus on the free energy landscape for dimerization of model collagen triple helices. This is directly relevant to the cases where triple helices have been shown to form dimers in a biological context. However, it is also the first step in understanding the interactions stabilizing higher-order collagen assemblies, as dimer formation is likely a step on the pathway to higher-order assemblies and because, together with effective many-body interactions, the pair interactions between triple helices should also form the basis for the stability of assemblies of more than two triple helices. As POG is the consensus repeat unit of all collagens and a motif known to stabilize triple helices, POG-repeat triple helices have long served as models for collagen structure formation. We therefore chose, as our first model system, the interaction between two helices composed of POG repeats (sequences in Fig. 2A). Naturally, considering all possible degrees of freedom in the association of these triple helices results in a large configurational search space, which would be challenging to thoroughly sample. To get an initial understanding of the preferred binding modes of a pair of helices, we conducted initial unbiased simulations of a pair of POG helices. Taking advantage of the 7/2 helical symmetry of POG peptides (57,68,69), we constructed an “infinite” periodic POG-repeat triple helix, with a (POG)_7_ asymmetric unit (Fig. 2, *H* and *I*). Such a construction has the advantage of eliminating boundary effects and potential terminal fraying of the triple helices, which would be almost inevitable in any small model system. This periodic triple helix was paired with an unconstrained (POG)_5_ triple helix with capped termini. Characterizing the binding in terms of the angle between the helical axes, we readily see that there are two dominant binding modes, parallel and antiparallel, with similar populations, and a much smaller population in which the helices are bound at right angles (Fig. 2 *G*).

### POG-repeat dimerization is driven by hydrogen bonds mediated through hydroxyproline

Given the results of this preliminary analysis and the consensus that natural collagen triple helices are associated in parallel or antiparallel configurations (Fig. 1), we chose to model ideal parallel and antiparallel geometries. We also note that both parallel and antiparallel configurations have been observed in POG-repeat crystal structures (69,70). Considering only parallel or antiparallel alignments of triple helices allowed us to use a setup involving a pair of “infinite” collagen triple helices, reducing the number of degrees of freedom to their relative displacement (Δ*x*, Δ*y*, and Δ*z* in Fig. 2, *H* and *I*), as well as the rotation of the second helix about its axis. Using the displacements Δ*x*, Δ*y*, and Δ*z* as collective variables for biasing, we performed WT-metaD to determine the free energy surface for the association of two periodic POG_7_ triple helices.

**Figure 3:**
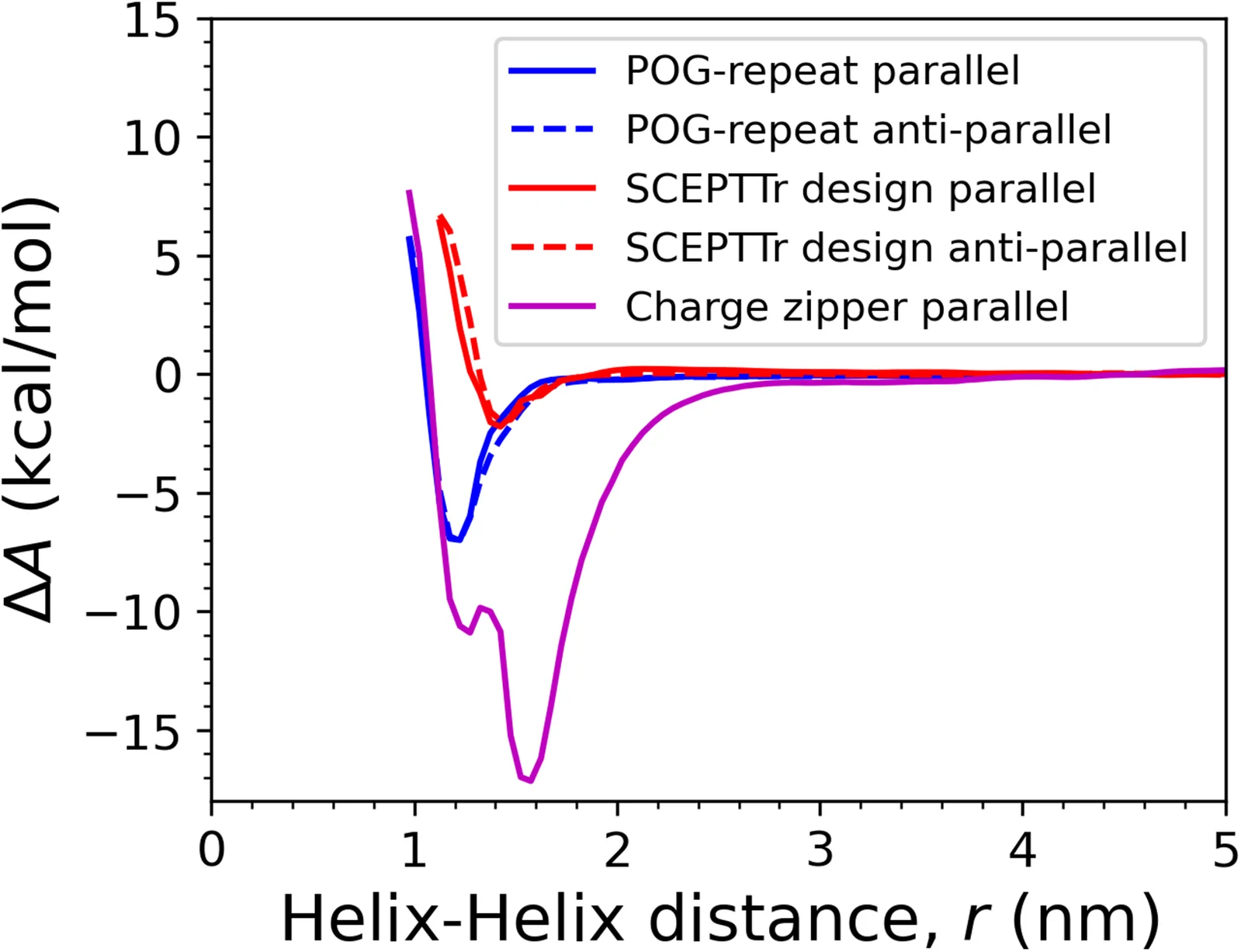
Free energy surface as a function of helix-helix distance for the five systems studied. Δ*A*(*r*) is determined by reweighting Δ*A*(Δ*x*,Δ*y*,Δ*z*) to a polar coordinate system, Δ*A*(Δ*r*,Δ*ϕ*,Δ*z*), and integrating over Δ*ϕ* and Δ*z*. The shallower free energy minimum in the charge zipper forms due to hydrophobic interfaces similar to those of the parallel POG repeat, and the global minimum forms due to interhelix salt bridges. The repulsive regime is not plotted in all systems, as the free energy approaches infinity.

**Figure 4:**
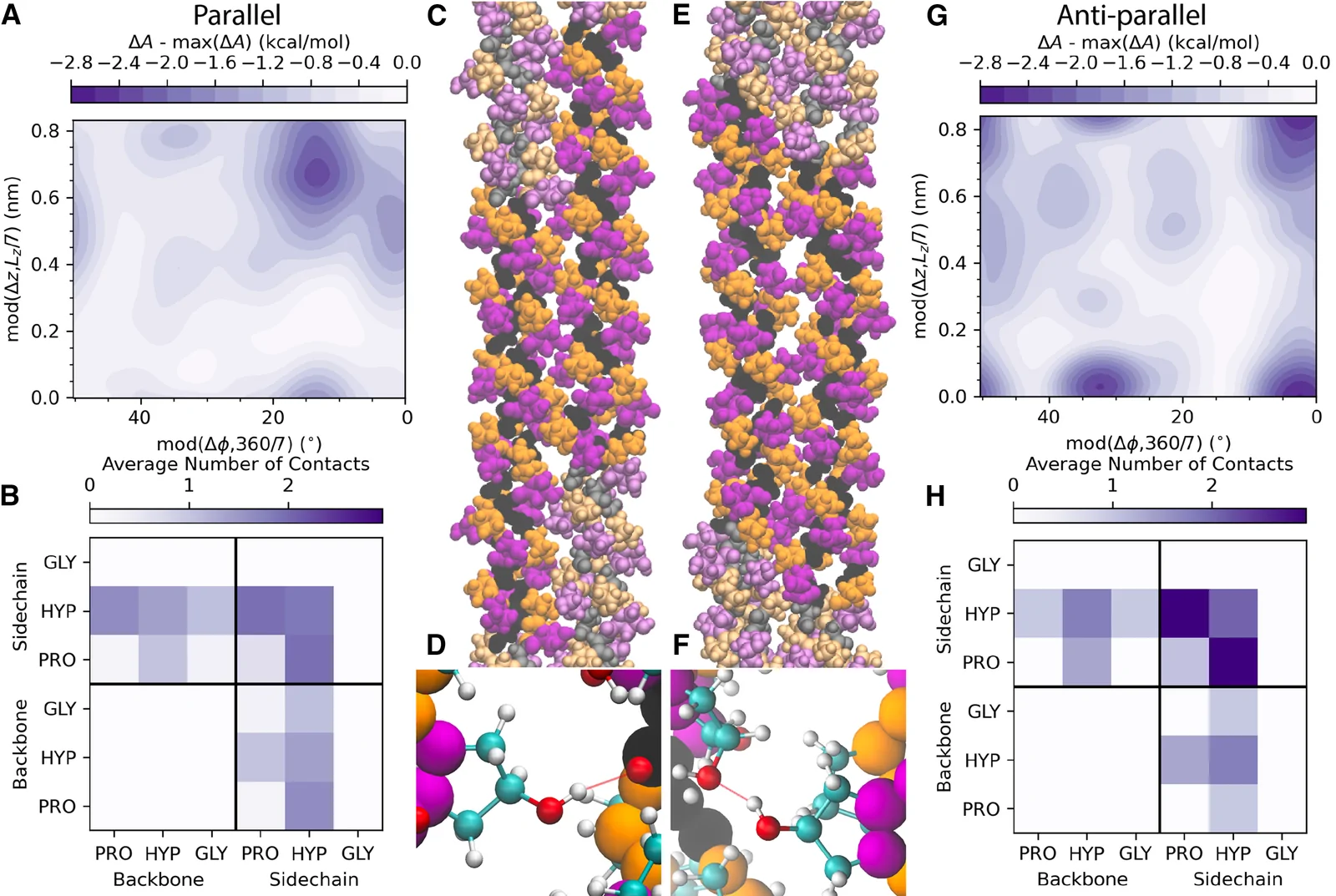
Association of POG-repeat triple helices in (*A–D*) parallel and (*E–H*) antiparallel configurations. Free energy surfaces of (*A*) parallel and (*G*) antiparallel POG-repeat triple helices symmetrized sevenfold over Δ*z*, and Δ*ϕ* (Δ*x* and Δ*y* are reweighted to angular azimuthal position). Average number of heavy atom backbone-backbone, backbone-side chain, and side chain-side chain contacts made within 4-Å distances in (*B*) parallel and (*H*) antiparallel triple helices between residue types. Minimum free energy conformations of (*C*) parallel and (*E*) antiparallel triple helices. The minimum at Δ*ϕ* = 2.5° is shown in (*E*). A Hyp–Gly hydroxyl-carbonyl hydrogen bond from the parallel conformation shown in (*D*). A Hyp–Hyp hydroxyl-hydroxyl hydrogen bond from the antiparallel conformation shown in (*F*). Pro, Hyp, and Gly amino acids are visualized in orange, purple, and black, respectively.

The potential of mean force (PMF) Δ*A*(*r*) for the association of a pair of periodic POG_7_ helices in Fig. 3 shows a helical diameter of 1 nm and a minimum located at around 1.2 nm with a depth of ∼7 kcal/mol, indicating a strong, favorable association of about ∼1 kcal/mol per POG repeat. Notably, the depth of this minimum is the same regardless of whether the helix orientation is parallel or antiparallel. To gain more insight into the nature of the interactions stabilizing this association, we have computed the PMF for a reduced set of coordinates, the relative shift of the helices along the *z* axis, Δ*z*, and the angular position of the second helix relative to the first in the *x*,*y* plane, Δ*ϕ*. Since, by construction, these 7-repeat sequences must have periodic features in *z* and in *ϕ* in their PMFs, we determined the free energy surface for the repeating unit. The PMF for the parallel orientation (Fig. 4 *A*) shows a single major minimum, characterized by contacts involving Pro and Hyp (Fig. 4 *B*), featuring a tight hydrophobic interface defined by contacts not only between Pro and Hyp side chains of opposing triple helices but also featuring contacts of side chains with the protein backbone (Fig. 4 *C*). The pyrrolidine rings of Pro and Hyp do not only participate in hydrophobic interactions, but the Hyp hydroxyls also participate in hydrogen bonding with backbone carbonyls and the Hyp hydroxyls of the opposing triple helix. We defined hydrogen bonds as formed within a ≤3.2 Å donor-acceptor distance and a ≥120° donor-hydrogen-acceptor angle. In the parallel configuration, the Hyp hydroxyl acts as a donor, forming 2.87 hydrogen bonds with the Gly carbonyl on average and 0.13 hydrogen bonds with the Hyp carbonyl. This specific Hyp–Gly hydrogen bond (Fig. 4 *D*) defines the single bound conformation, requiring the triple helices be axially displaced by ∼0.65 nm and rotated by 13° relative to each other.

On the other hand, the antiparallel configuration, as shown in Fig. 4, *E–H*, shows two clear minima (Fig. 4 *G*), distinguished by the relative rotation of triple helices by 2.5° and 32.5°. In the antiparallel configuration, the Hyp hydroxyl acts as a donor, forming 1.92 hydrogen bonds with the Gly carbonyl on average. However, the Hyp hydroxyl also acts as a hydrogen bond acceptor in this case, forming 0.86 hydrogen bonds (Fig. 4 *F*), as well as a higher average propensity to form 0.266 hydrogen bonds with the Hyp carbonyl group. These two slightly weaker hydrogen-bonding patterns seem to contribute to the apparent intercalation of Pro and Hyp at gaps between the 14 positions Hyp and Pro can occupy in the 7/2 helix (71), such that Pro-Hyp interactions appear more frequently in side chain-side chain interaction contact maps (Fig. 4 *H*).

The POG-repeat dimers we found are somewhat different from the crystal structures of POG repeats, which feature a lamellar structure with planes of parallel and antiparallel POG triple helices (69,70). In the (GPO)_9_ crystal, each triple helix forms 4 and 3 Hyp–Hyp hydrogen bonds between the hydroxyl groups of two neighboring triple helices with which it is parallel, adopting an interhelical distance of 1.4 nm. Between the parallel and antiparallel triple helices also lie crystallographic waters. In dehydrated X-ray powder diffraction experiments, it was found that the interhelical distance of POG and PPG repeats can be as low as 1.2 nm (70) at 10% humidity. Thus, triple helices seem competent for forming stable interactions that are not mediated by water, provided the triple helix is formed by residues that can adopt such a tight hydrophobic packing. Leikin et al. had also observed a hard upper limit in osmotic force in helix-helix spacing approaching 1.2 nm, implying that dehydration is necessary to form such a tight dimer (72). It seems that POG or PPG-repeat triple helices in aqueous conditions must undergo a transition from dimeric to oligomeric or fibrillar states when encountering additional triple helices in solution to adopt a significantly different set of atomic interactions between helices mediated by collagen-solvent interactions.

### Designed intrahelix salt bridges do not break to form interhelix salt bridges

Although POG may be the consensus building block of collagen, natural collagens generally do not have multiple consecutive POG repeats but do contain substitutions at the X and Y positions in the XYG repeat. These substitutions can affect both triple helix stability as well as the association of helices. In particular, interactions between side chains at these positions, such as salt bridges or cation-*π* interactions, can be used to engineer stable collagen-mimetic triple helices. As a recent exemplar of such stabilized helices, we have chosen a designed triple helix with above-physiological thermal stability (melting temperature: ∼313 K) designed using the knowledge-based SCEPPTr scoring function (30) (Fig. 2 *C*). A notable feature of this design is the inclusion of multiple salt bridges stabilizing the triple helix, as indicated schematically in Fig. 2 *D*, in addition to other stabilizing interactions. WT-metaD was again used to determine the free energy surface for association.

**Figure 5:**
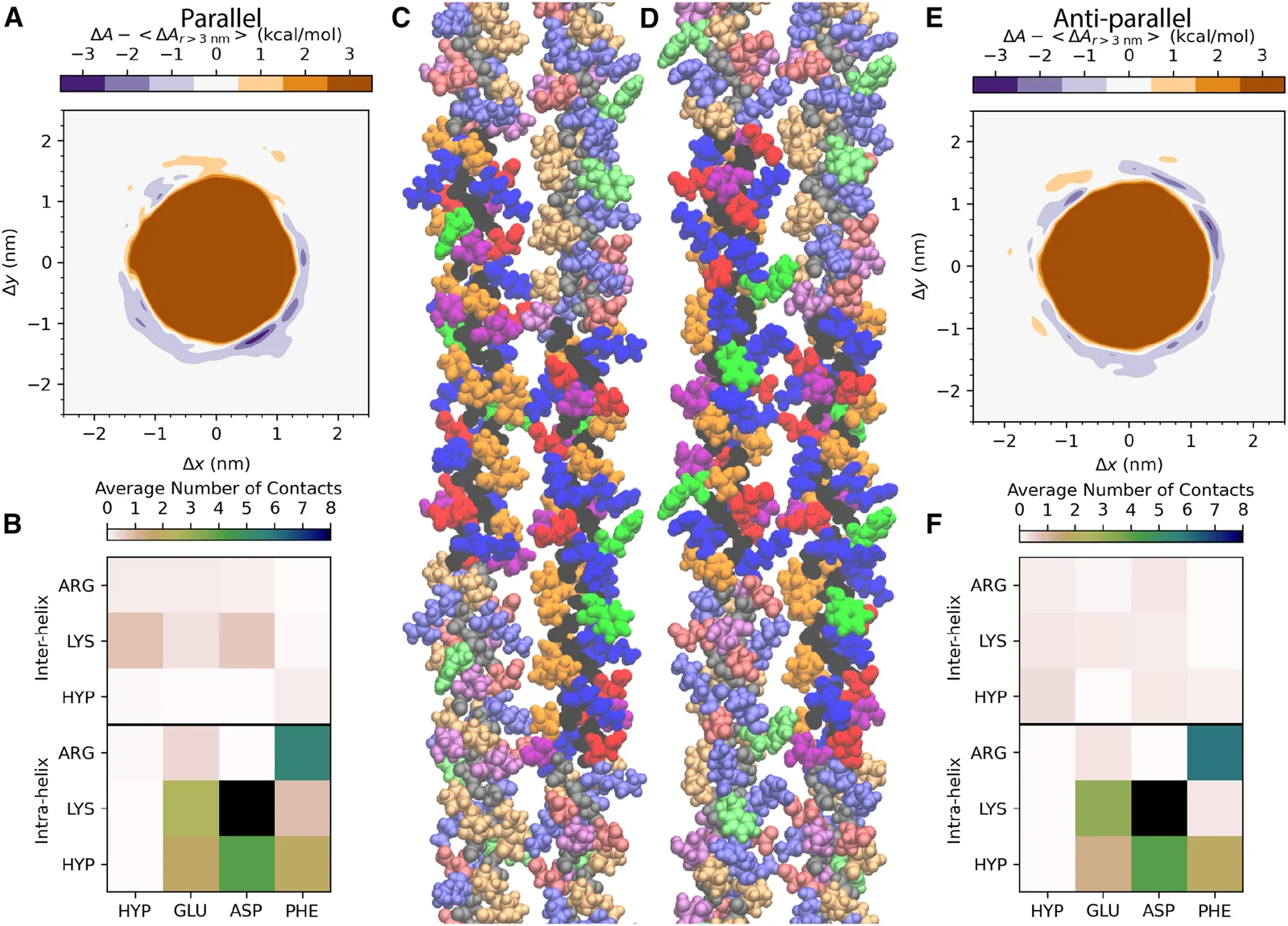
SCEPPTr-designed triple-helix systems in (*A–C*) parallel and (*D–F*) antiparallel orientations. Free energy surfaces integrated over *Δz* for (*A*) parallel and (*E*) antiparallel SCEPPTr-designed triple helices, showing very shallow binding interfaces. Average number of interhelical and intrahelical heavy atom pair contacts within 4-Å in (*B*) parallel and (*F*) antiparallel triple helices between residue types. Representative configurations at minimum free energy in (*C*) parallel and (*D*) antiparallel are colored with Arg and Lys amino acids in blue, Glu and Asp amino acids in red, and Pro, Hyp, Gly, and Phe amino acids in orange, purple, black, and green, respectively.

Remarkably, we observe only a very small minimum in the association PMF of ∼2 kcal/mol at a helix-helix distance of ∼1.4 nm, and a similar weak association is found for both parallel and antiparallel orientations (Fig. 3). The shift in position of the minimum could be understood as arising from the more bulky side chains; however, the weak association was not expected, given the known importance of salt bridges for stabilizing interactions between triple helices in natural collagen. Computing the free energy landscape using the displacement coordinates Δ*x*,Δ*y* of the second triple helix from the first confirms a near-flat free energy landscape, with a few shallow minima, as seen in Fig. 5, *A,E*. Analysis of the contact formation frequencies (determined from the underlying probability density function) shows that, on average, very few contacts are formed between triple helices; by contrast, close to the maximum possible number of designed intrahelix salt bridges and cation-*π* interactions are observed (Fig. 5, *B,F*).

These results demonstrate, on the one hand, the success of the design in stabilizing intrahelical interactions but also highlight that the availability of multiple charged residues does not necessarily mean that salt bridges will form between the helices. In this case, the sequence has been designed to facilitate cooperative formation of salt bridges in the target triple-helix structure, and the formation of salt bridges between helices would very likely lead to the frustration of both intra- and intermolecular salt-bridge interactions.

Additionally, we note that this demonstrates how the presence of charged and polar residues can also occlude the surface of the triple helix from participating in the type of tight hydrophobic interactions seen in POG-repeats. The salt bridges that stabilize the SCEPPTr-designed sequence studied here, for example, effectively expanded the triple-helix diameter from 1 to 1.2 nm, abolishing the possibility of forming a tight dimer complex (Fig. 3). Models of helix-helix interactions that include hydrophobic interactions may need to consider that such interactions may be reduced or abrogated by the neighboring residues in the triple helix.

### Interhelix salt bridges can strongly stabilize interhelix interactions

To test for the potential effect of salt bridges on triple-helix association, we designed a pair of triple helices so as to preclude the formation of intrahelix salt bridges and promote the formation of interhelix salt bridges. These sequences are shown in Fig. 2 *E*, and the potential interhelix salt bridges are depicted in Fig. 2 *F*. This pair of triple helices was designed as a model case in which a dimer of triple helices would form along a specific pair of helix faces. To ensure this, we designed a charge zipper model, in which the Y-position amino acids occupied by Hyp in POG-repeat triple helices are substituted with Lys or Glu at nine sites, such that there are three faces of the triple helices that are occupied by alternating positive or negatively charged residues that can zip up together in a manner evocative of the joining of two tapes of a zipper, held together by interlocking teeth. The remaining, unsubstituted POG triplets would remain available for a weaker helix-helix interaction on the order of that observed in the characterization of POG-repeat helix-helix dimers.

**Figure 6:**
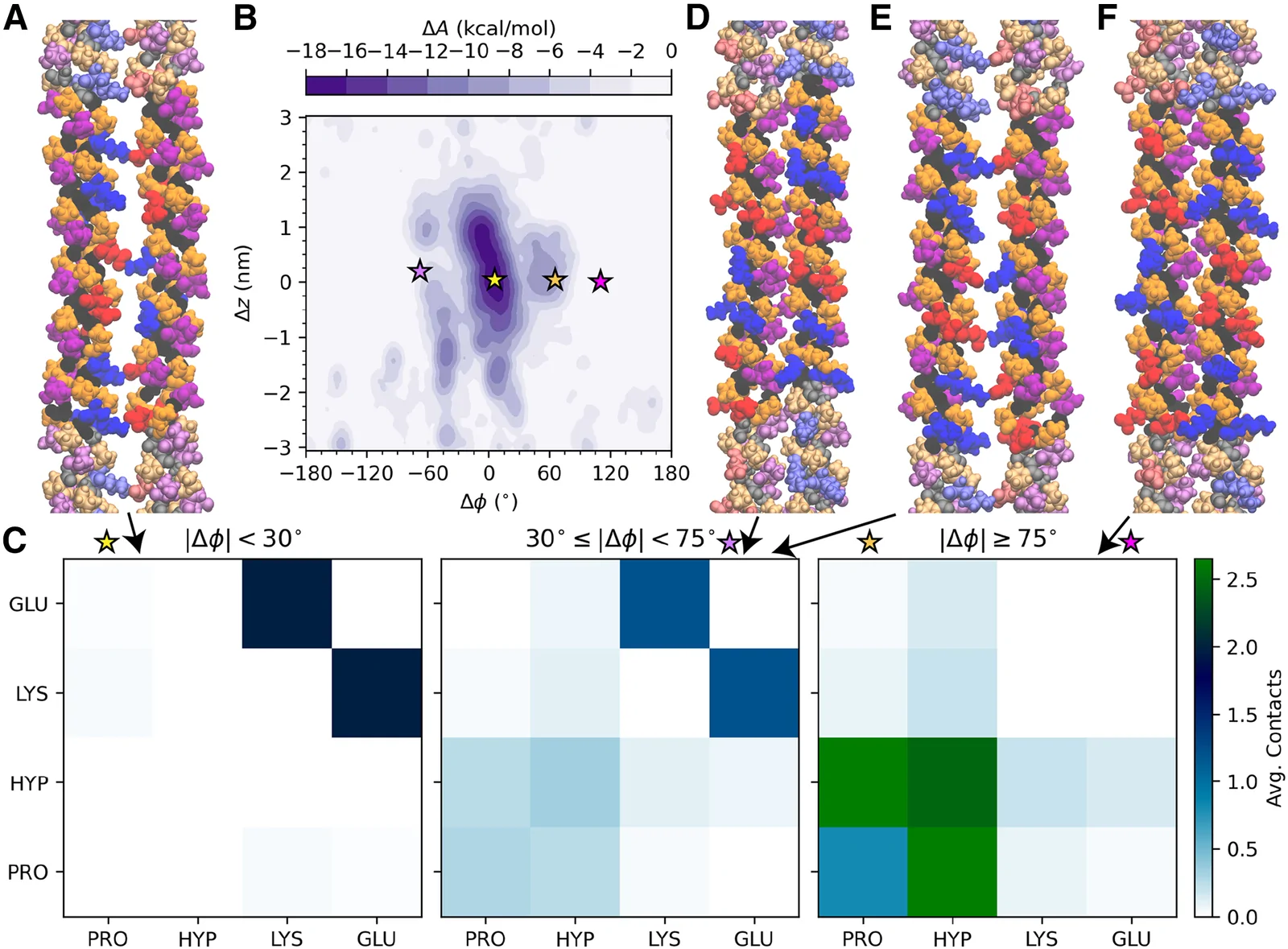
Association of designed charge zipper sequences. (*A*) Charge zipper heterotrimer at free energy minimum. (*B*) Free energy surface reweighted to Δ*A*(Δ*ϕ*,Δ*z*), where Δ*ϕ* is the azimuthal angular position. (*C*) Number of residue-residue heavy atom contacts within 4-Å between triple helices in three bins, demonstrated with representative configurations at (*A*) |Δ*ϕ*| < 30° (*yellow star*), (*D*) side-on interactions in 30° < |*ϕ*| < 75° (*purple star*), (*E*) salt bridge-salt bridge interactions in 30° < |*ϕ*| < 75° (*orange star*), and (*F*) hydrophobic interactions driven by Hyp–Hyp hydrogen bonds in |*ϕ*| ≥ 75° (*magenta star*). Pro, Hyp, Gly, Lys, and Glu amino acids are colored in orange, purple, black, blue, and red, respectively.

The free energy for association of these helices shows two minima when computed as a function of the interhelix distance: a primary minimum at ∼1.55 nm and an energy of −17 kcal/mol and a secondary minimum at a similar distance to the minimum for the POG_7_ helices ∼1.2 nm, which has an energy of −11 kcal/mol. We visualize the free energy surface of the associating helices in a Δ*ϕ*,Δ*z* representation in Fig. 6. The main free energy minimum indeed corresponds to the idealized interaction of the two helices that correspond primarily to salt-bridge interactions (Fig. 6, *A,C*). Given an average of ∼4 salt bridges formed in the complex, the free energy per salt bridge is ∼4 kcal/mol, in line with other estimates for this energy scale (73). The subsidiary minima show evidence of being stabilized by both salt bridges and the shorter-range hydrophobic and hydrogen-bonding interactions that stabilize the POG repeats (Fig. 6, *C,E*). Some low-populated minima are stabilized primarily by POG-like interactions, without formation of any salt bridges (Fig. 6, *C,F*). Overall, the picture is one in which potential salt bridges that are able to easily form in concert can greatly stabilize collagen dimers, more than doubling the free energy of association relative to the simple POG-repeat prototypes. At the same time, when all the salt-bridge interactions are located on one side of the triple helix, as is done here, it remains possible to form shorter-range POG-like interactions that were not possible in the SCEPPTr-designed sequence, either with or without simultaneous formation of the salt bridges. Thus, the emergence of multiple minima for the charge zipper sequence seems to be a consequence of the ability of this sequence to access both the hydrophobic interactions at shorter triple-helix separations as well as salt-bridge interactions at longer separations, which is not possible for the POG and SCEPPTr-designed sequences.

### Approximately one-fourth of charged residues in fibrillar collagens may be unavailable for interactions with other proteins

We observed that the SCEPTTr-designed sequence, in which the triple helix is stabilized by charged residues and cation-*π* interactions (Fig. 2, *C* and *D*), does not release charged residues from these intrahelical interactions to engage in homodimerization. This is quite interesting, as it has been observed that the fibril diameter (74) reaches a maximum around 150 mM salt, and the fibrillization rate is substantially slower than in neat water and faster than in 300 mM salt (75,76), suggesting an important role for interactions between charged residues. Theoretical models of collagen dimerization and fibrillization have relied on this observation for simplified models of fibrillogenesis and *D*-band formation, assuming that all charged residues participate in interactions between triple helices (77,78). Although some models have also assumed that hydrophobic interactions involving Hyp and possibly aromatic residues can contribute favorably to fibrillogenesis as well (79,80), there are few aromatic residues in fibrillar collagens, with the most and least appearing at positions with ≥80% frequency in MSAs in collagen II and collagen III, having 14 and 8 residues per strand, respectively. Moreover, Hyp-Hyp interactions are shorter range than charge-charge interactions.

Given this observation, it may be necessary to consider the effect of intrahelix salt bridges on models of fibrillogenesis driven by charged residue association. The simplest way of doing this is to remove from consideration those charged residues that are positioned to interact via helix-stabilizing axial or lateral salt bridges formed by Lys-Glu or Lys-Asp pairs (81). Such salt bridges have become central to designing stable triple helices (82). Although fibrillar collagen triple helices are not thermally stable in the absence of disulfide cross-linked C-propeptide domains or the HSP47 chaperone (83,84), it has been proposed that the significant kinetic stability endowed by Lys-Glu and Lys-Asp salt bridges enables the triple helix to remain folded during the secretion process and the initial stages of collagen fibrillization (65).

To analyze how many Lys, Glu, and Asp residues are typically engaged in axial (Fig. 2 *D*) or lateral salt bridges and the number of Lys, Arg, Glu, and Asp otherwise “free” to participate in helix-helix and other protein-collagen interactions, we took the following approach. We developed MSAs of all fibrillar collagen strands, identified positions where ≥80% of amino acids are Lys (or Arg) and Glu or Asp, and analyzed whether Lys and Glu or Asp positions are arranged to form an axial or lateral salt bridge (see Jalan et al. for a review on helix-stabilizing salt bridges (85)). After removing those residues from consideration, we determined the number of remaining Lys, Arg, Glu, and Asp available to participate in other interactions (Table 1). We found that approximately equal numbers of Lys, Glu, and Asp participate in axial and lateral interactions and that approximately one-fourth of all charged residues participate in these interactions. There are 368–438 charged residues in these triple helical domains that remain available for participating in helix-helix and other helix-protein interactions. The 107–201 charged residues that participate in helix-stabilizing axial or lateral interactions may be less available to participate in fibrillization or other protein-protein interactions involving the triple-helix domain and may have evolved primarily to stabilize the triple helix. Although one may imagine a scenario in which there are arrangements for forming either intramolecular salt bridges or an alternative set of intermolecular salt bridges with a similar degree of cooperativity, this seems unlikely. To fully explore the relative contributions of inter- and intramolecular salt bridges will likely require a simulation or statistical mechanical model in which both possibilities are allowed.

**Table 1:** Analysis of charged residues at over ≥80% occupancy at positions in triple helices identified from multiple sequence alignments of the fibrillar collagens I, II, III, V, and XI

Helix assembly	Charged residues	K, D, and E in axial Int.	K, D, and E in lateral Int.	Free K, R, D, and E
1*α*1,1*α*1,1*α*2	475	62	52	368
(2*α*1)_3_	519	76	62	392
(3*α*1)_3_	444	78	66	312
5*α*1,5*α*2,5*α*3	557	66	70	438
11*α*1,11*α*1,11*α*2	599	114	120	398

In proposed triple-helix assemblies, intrahelix salt bridges formed by axial or lateral K–E or K–D interactions among conserved charged residues are counted. Remaining charged residue positions available for interhelix and other collagen-protein interactions are counted as “free.” A small number of sites may participate in both axial and lateral charged interactions.

## Conclusion

By considering the association of several model collagen triple helices, we have observed association energy landscapes that are strongly sequence dependent, with subtle features that may not have been anticipated from a simple consideration of sequence composition. First, POG repeats exemplify a fundamental sequence motif which is available to participate in the association of any naturally-occuring collagen sequence, driven by hydrohopbic interactions and stabilized by hydroxyproline hydrogen bonds. This mode of interaction occurs at a short distance of ∼1.2 nm and can contribute ∼1 kcal/mol per POG repeat to the association free energy. Naturally occurring collagen helices are known to associate via charged interactions, and we have found that model salt-bridge interactions typically occur at a larger distance of ∼1.6 nm, with a stabilization of ∼4 kcal/mol per salt bridge. However, the different distance ranges corresponding to these interactions can potentially lead to frustration. In our designed charge zipper model, in which charged residues lined only one side of the helix, it was possible to form both types of interactions in the same system, albeit corresponding to different relative orientations of the helices. For both peptides, the effective repulsive radius was ∼1 nm. In the other example considered, the SCEPPTr-designed sequence, the triple helix was surrounded on all sides by bulkier residues, so the shortest accessible distance was ∼1.2 nm regardless of helix orientation. That example highlighted another complexity, namely apparent frustration between inter- and intrahelix salt bridges: the sequence had been optimized for intrahelical salt bridges to stabilize the triple helix so that virtually no interhelical salt bridges were formed, resulting in a small association free energy. A further intriguing result was that parallel and antiparallel association free energies were very similar for each of the POG and SCEPPTr-designed sequences.

To extend the observation that intrahelix salt bridges involving Lys may preclude the formation of interhelix salt bridges in these model systems to natural collagen, we have analyzed sequences of triple helices formed by the fibrillar collagens. We found that approximately one-fourth of all charged residues in these collagens are positioned to form intrahelix salt bridges and thus may be unavailable for interactions with other proteins in extracellular matrices or for participation in fibrillogenesis. This observation may be critical for the parameterization of statistical mechanical models of fibril structure, which hitherto have considered all charged residues as equal contributors to helix-helix interactions.

The subtle effects captured in the association free energy surfaces of these model peptides justify our choice of all-atom simulations to perform this study. Nonetheless, even a single full-length collagen triple helix is a very large system, and so coarse graining is essential for describing the large-scale assembly mechanism of collagen fibers. The present study will help to inform the design of coarse-grained models that can best capture some of the important complexities that we have identified in this work, with the goal of a more complete description of collagen self-assembly.

## Data and code availability

Simulation inputs and selected project data are available on Zenodo at https://doi.org/10.5281/zenodo.18133224.

## Acknowledgments

G.A.P. and R.B.B. were supported by the Intramural Research Program of the 10.13039/100000062National Institute of Diabetes and Digestive and Kidney Diseases (NIDDK) within the 10.13039/100000002National Institutes of Health (NIH). The contributions of the NIH authors are considered works of the United States government. The findings and conclusions presented in this paper are those of the authors and do not necessarily reflect the views of the NIH or the US Department of Health and Human Services. J.E.S. and A.M. gratefully acknowledge the generous support of the 10.13039/100000001National Science Foundation (CHE1900416) and the NIH (R01 GM107703). This work utilized the computational resources of the NIH HPC Biowulf cluster (https://hpc.nih.gov).

## Author contributions

G.A.P., A.M., J.E.S., and R.B.B. designed and discussed the research. G.A.P. carried out all simulations and data analyses. G.A.P. and R.B.B. wrote the article.

## Declaration of interests

The authors declare no competing interests.

## Supporting material

Document S1. Figures S1 and S2 and Table S1

Data S1. Supplemental information including MSA lengths and depths, convergence of free energy as a function of helix-helix distance, and final free energy surfaces in terms of *x*- and *y*-displacements of helices integrated over *z*-axial displacements

Document S2. Article plus supporting material
