# Supplementary material for: Free energy of collagen-mimetic peptide dimerization and implications for fibrillization
Source: Biophys J. 2026 Jan 13;125(16):4192–203. doi: 10.1016/j.bpj.2026.01.010 (PMC13507419; doi:10.1016/j.bpj.2026.01.010)
Supplement: Supplementary file 1 [file mmc1.pdf]

Biophysical Journal, Volume *125*

Supplemental information

Free energy of collagen-mimetic peptide dimerization and implications for fibrillization

George A. Pantelopulos, Ayan Majumder, John E. Straub, and Robert B. Best

Manuscript submitted to **Biophysical** *Journal*

Supporting Information for

Free Energy of Collagen-Mimetic Peptide Dimerization and Implications for Fibrillization

George A. Pantelopulos[1,*], Ayan Majumder[2], John E. Straub[2], and Robert B. Best[1,*]

[1]Laboratory of Chemical Physics, National Institute of Diabetes and Digestive and Kidney Diseases, National Institutes of Health, Bethesda, Maryland 20892-0520, USA
[2]Department of Chemistry, Boston University, Boston, Massachusetts 02215-2521, USA
[*]Correspondence: george.pantelopulos@nih.gov, robert.best2@nih.gov

Table S1: Length and number of sequences in multiple sequence alignments of the fibrillar collagens. These MSAs were used for analyzing the number of inter-strand salt bridges formed among sequence positions where Lys, Arg, Glu, or Asp residues appear with ≥80% frequency.

Collagen Type	Length	Num. Seq.
1A1	1017	462
1A2	1017	689
2A1	1017	505
3A1	1020	402
5A1	1014	709
5A2	1017	513
5A3	1014	396
11A1	1014	931
11A2	1014	1047

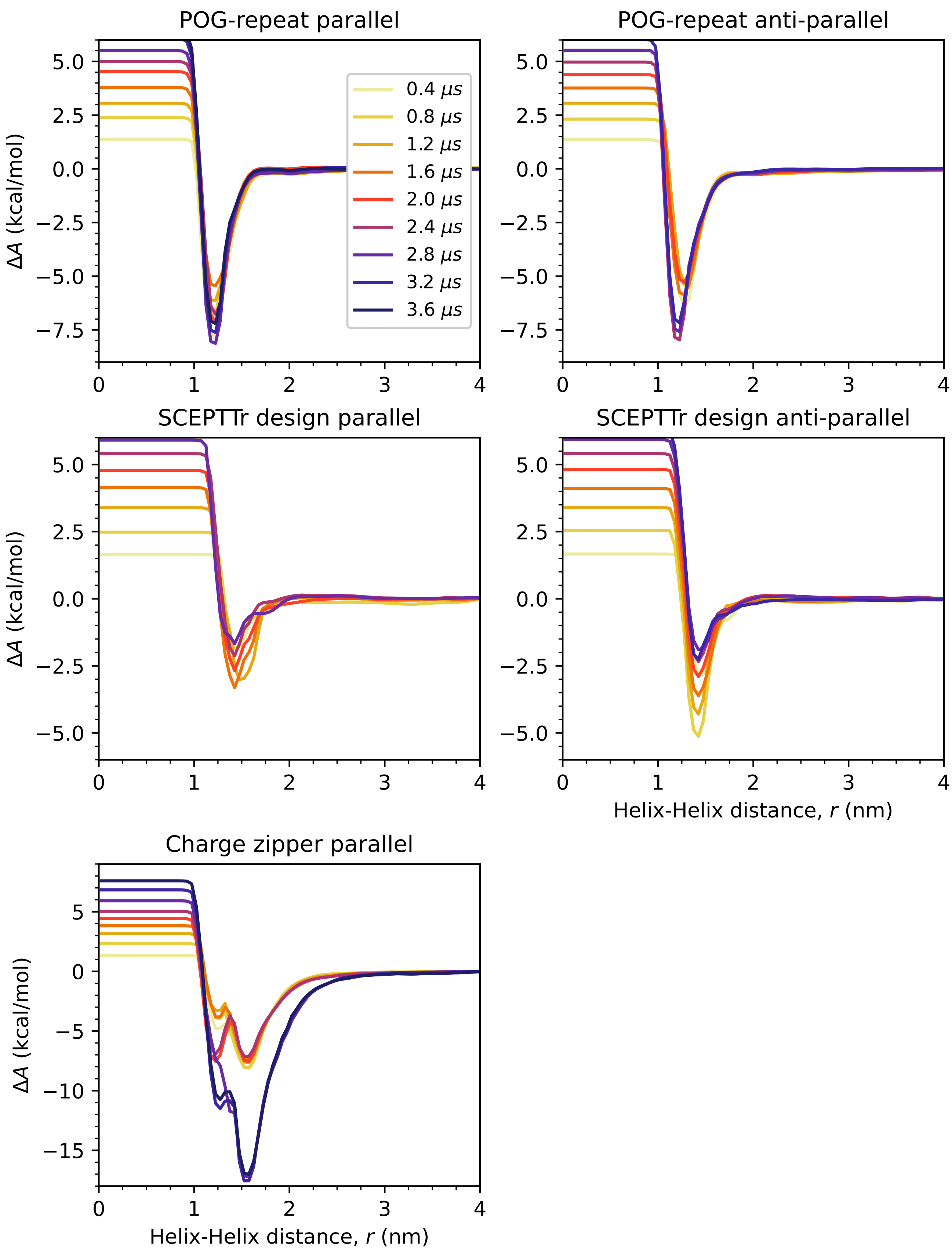

Figure S1: Free energies as a function of distance between centers of triple helices as WT-metaD traejectories progress over the course of time simulating each of 12 parallel trajectories. The characteristic free energy minima within each system appear to close to convergence after 2.8 μs.

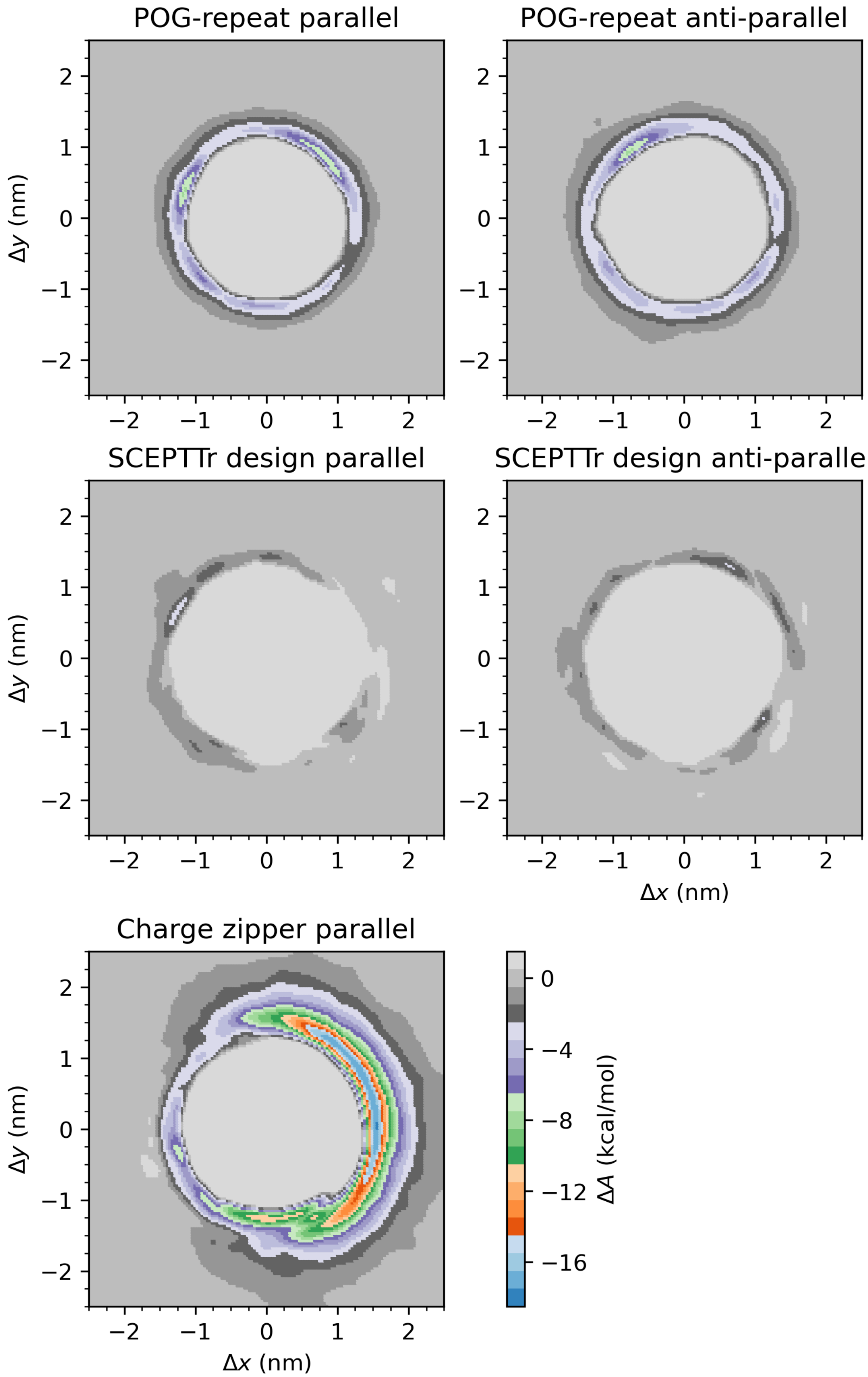

Figure S2: Final free energy surfaces represented in $\Delta x \Delta y$-plane for the five CMP systems studied via WT-metaD.
